# In Syria, Unqualified People are Renting Pharmacists’ Licenses to Open Drugstores: A Phenomenon that Threatens Public Health

**DOI:** 10.3389/ijph.2022.1605224

**Published:** 2022-11-28

**Authors:** Mohamad Saleem Anis

**Affiliations:** Discipline of Social and Administrative Pharmacy, School of Pharmaceutical Sciences, Universiti Sains Malaysia, Penang, Malaysia

**Keywords:** public health, ethics, pharmacy practice, pharmaceutical policy, pharmacy degrees rental, Syria

**The IJPH series “Young Researcher Editorial” is a training project of the Swiss School of Public Health.**

The pharmacy profession in Syria and other countries, including Pakistan, Cambodia, Vietnam, and Yemen, has been harmed by the widespread practice of non-pharmacists renting a pharmacist’s professional license (1–3). In Syria, the problem usually begins when pharmacy students graduate and face barriers to entering the market, such as the considerable sum of money needed to start a private community pharmacy (4). Graduates often resort to the easier solution of renting out their license to any financially capable person, even if they hold no medical qualification. Though common, this act flagrantly violates the applicable law.

The Syrian Law of Practicing Medical Professions limits the right to open a pharmacy to a registered pharmacist, who must also serve as the manager (5). Renting, lending, or any transfer of rights, privileges, and responsibilities of pharmacists is illegal because non-pharmacists are ignorant of national drug regulations and may traffick in smuggled and substandard medicines. If those who sell drugs do not know the pharmacology of each drug and cannot properly advise customers on its use, license rental can have serious consequences.

The phenomenon of license rental has multiple underlying reasons. Pharmacists may rent out or lend their licenses for reasons that vary from case to case, but several reasons are prominent. First, many new pharmacy graduates want to avoid the 2-year period of mandatory rural service because they do not want to travel outside their home city. They thus allow a renter to run a pharmacy in a rural area under their name, giving them use of their “temporary practice permit,” for an agreed monthly fee or other benefits. Second, traditions can influence this decision. Traditionally, it is often difficult for a woman to work alone in a pharmacy, so her husband or another person works under her name and pays monthly rent for her license. Third, the problem has been exacerbated by war. Many pharmacies were partially or fully destroyed in hot spots, and most owners considered renting their licenses to others so that they could continue to earn income under these conditions.

The nation’s private universities are graduating an increasing number of pharmacists, driving up supply. In 2018, there were over 30,000 registered pharmacists in Syria (6). Growing imbalance between supply and demand has increased the number of unemployed pharmacists, especially recent graduates with limited financial resources. Since 2013, the sharp decline in the value of local currency has made it harder for pharmacists to open their own pharmacies, so many lease their licenses to others and also earn income working elsewhere.

Renting out pharmacists’ licenses harms public health. In Syria, where pharmacists are frequently referred to as “doctors of the poor,” an incompetent pharmacy owner who has little scientific knowledge may endanger the lives of citizens. They may make dispensing errors, diagnostic errors, or provide incorrect information to patients about drugs, drug interactions, and addictive substances.

When untrained persons assume the pharmacists’ position in pharmacies, it shakes public confidence in real pharmacists. The medical errors made by those who impersonate pharmacists have caused citizens to lose confidence in all pharmacists. Under current conditions, they do not know if the person who runs the pharmacy holds a pharmacy degree or not.

The pharmacy profession is expected to provide high-quality patient care, especially in the case of community pharmacies [7]. But those who rent pharmacy licenses are mainly focused on profits rather than offering the most effective and affordable treatments to patients.

Pointing out and identifying any problem is the first step towards solving it, even when the root causes are systemic and thus not easy to control. If early-career pharmacists rent out licenses because they feel they have no other options, it should be possible to create and test a short-term implementation plan for them with, for example, zero-interest loans, while also pursuing pragmatic approaches to monitoring and enforcing the existing law that prevents license rental. To accomplish these ends, there is urgent need for governmental and related community organizations to move quickly to minimize the spread of license rental and ultimately prevent it.
